# *Aspergillus neoalliaceus* MR-86 Promotes the Growth of *Saposhnikovia divaricata* by Regulating the Rhizosphere Microbiome

**DOI:** 10.3390/plants15111703

**Published:** 2026-05-31

**Authors:** Yanzhe Ding, Yuqi Zhao, Yuanxin Xie, Chongbo Sun, Limin Yang, Zhuo Sun, Li Yang, Yunhe Wang, Jian Zhang, Zhongming Han

**Affiliations:** 1 Jilin Provincial Key Laboratory of Ecological Cultivation of Medicinal Plants, Key Laboratory of Ecosystem and Ecological Restoration of Jilin Province, College of Chinese Medicinal Materials, Jilin Agricultural University, Changchun 130118, China; ding18043426173@outlook.com (Y.D.); 15044591690@163.com (Y.Z.); 15943437796@163.com (Y.X.); ylmh777@126.com (L.Y.); zhuos@jlau.edu.cn (Z.S.); yangliff@126.com (L.Y.); 2 Horticulture Institute, Zhejiang Academy of Agricultural Sciences, Hangzhou 310021, China; suncb@zaas.ac.cn; 3 Department of Biology, University of British Columbia, Okanagan, Kelowna, BC V1V1V7, Canada

**Keywords:** *Saposhnikovia divaricata*, *Aspergillus neoalliaceus*, plant growth-promoting traits, physicochemical properties, enzyme activities, rhizosphere microbiome

## Abstract

Plant growth-promoting fungi (PGPF) have shown broad potential to improve soil conditions and enhance root growth and development. However, few studies have examined the effects of exogenous PGPF inoculation on the growth of the medicinal plant *Saposhnikovia divaricata* and the associated changes in the rhizosphere microbiome. In this study, *Aspergillus neoalliaceus* MR-86 exhibited phosphate solubilization, growth in nitrogen-free medium, potassium solubilization, IAA production, and siderophore production. PCR assays did not detect the aflatoxin biosynthesis-related genes *aflR*, *aflS*, and *omtA* in strain MR-86. Pot trials demonstrated that inoculation with MR-86 significantly increased the plant height and root dry weight of *S. divaricata* by 10.32% and 21.05%, respectively (*p* < 0.05). In the rhizosphere, soil pH decreased, whereas soil alkaline-hydrolyzable nitrogen and available phosphorus levels, as well as the activities of protease, urease, and cellulase, increased significantly. Illumina NovaSeq sequencing revealed that MR-86 inoculation altered the soil microbial community structure and specifically enriched several microbial taxa, including *Talaromyces*, *Subulicystidium*, and *Aspergillus*. Moreover, MR-86 inoculation did not alter the composition of dominant bacterial and fungal phyla, but significantly modified microbial interactions and the topology of microbial networks. Correlation analysis indicated that the specific microbial taxa *Subulicystidium*, *Aspergillus*, and *Talaromyces* were positively associated with soil nutrient indices, enzyme activities, and plant growth parameters. Functional prediction analysis indicated that MR-86 treatment was predicted to be enriched bacterial metabolic pathways, including flavone and flavonol biosynthesis and ether lipid metabolism, and was predicted to increase the relative abundance of functional fungal groups such as ectomycorrhizal and wood-decomposing fungi. In summary, *A. neoalliaceus* MR-86 may contribute to improved growth of *S. divaricata* by enhancing nutrient availability and transformation and by modulating the structure and function of the rhizosphere microbiome.

## 1. Introduction

The rhizosphere microbiome, which is closely associated with the root surface and surrounding soil [1,2], constitutes a second genome of plants and plays crucial roles in plant growth and development. These microorganisms contribute to nutrient cycling, regulate plant growth, and suppress pathogenic microbes [3,4], thereby playing key roles in plant development and stress adaptation [5]. In recent years, the use of beneficial microorganisms with specific functions, such as plant growth-promoting fungi (PGPF) and plant growth-promoting rhizobacteria (PGPR), to regulate the structure and function of rhizosphere microbial communities has been recognized as an important strategy for improving soil quality, increasing crop productivity, and achieving sustainable agricultural development [6,7,8]. These beneficial microbes can enhance nutrient availability and transformation, regulate the rhizosphere microecological environment, and ultimately promote plant growth [9,10].

In current agricultural systems, chemical fertilizers are widely used. However, their long-term excessive application can accelerate the mineralization of soil organic matter, leading to the depletion of soil carbon pools and nutrient imbalances [11]. Therefore, developing sustainable agricultural strategies to reduce reliance on chemical fertilizers is urgently needed. In this context, plant–microbe symbiotic systems have attracted considerable attention as environmentally friendly and agriculturally sustainable approaches. Such systems not only enhance plant nutrient uptake but also improve the balance of the soil microecological environment, thereby promoting crop growth and yield while reducing dependence on chemical inputs. With advances in green agriculture, increasing efforts have been devoted to the artificial inoculation of beneficial rhizosphere microorganisms to modulate native microbial communities [12,13], thereby improving crop production and reducing fertilizer inputs. Numerous studies have reported that PGPR and PGPF can enhance nutrient utilization, increase microbial community diversity, and promote the abundance of beneficial microbes, thereby promoting plant growth [14,15,16].

*Saposhnikovia divaricata*, a medicinal plant in the family Apiaceae, is widely distributed in northeastern China and Inner Mongolia. The dried root of this plant, harvested before bolting, is used as a traditional medicinal material and represents an important resource in Chinese herbal medicine. It is a key component of the traditional Chinese patent medicine Fangfeng Tongsheng Pills [17], and has been reported to possess various pharmacological activities, including anti-inflammatory, analgesic, anti-rheumatic, anticancer, and anti-allergic effects [18,19]. However, overharvesting has led to a decline in wild populations [20], and cultivated *S. divaricata* has become the primary source of medicinal materials [21]. However, improving yield and soil quality through ecological regulation during cultivation remains a significant challenge. In recent years, research on the application of PGPF in agriculture has increased substantially, with *Trichoderma* species being the most extensively studied [22,23,24]. In contrast, fungi of the genus *Aspergillus*, although known for their abilities in phosphate solubilization, organic acid production, and plant growth promotion [25,26], have been relatively underexplored in medicinal plants, particularly in *S. divaricata* cultivation.

In this study, *S. divaricata* was selected as a model medicinal plant, and the fungus *Aspergillus neoalliaceus* MR-86, a strain previously isolated from the rhizosphere of healthy *S. divaricata* by our research group, was used for inoculation experiments. This strain has not been investigated in previous studies. Whether *A*. *neoalliaceus* MR-86 can promote *S. divaricata* growth by reshaping the rhizosphere microbiome and improving soil nutrient availability remains unclear. Therefore, the effects of this strain on plant growth parameters, rhizosphere soil physicochemical properties, enzyme activities, and the structure and function of rhizosphere bacterial and fungal communities were systematically investigated. This study aims to provide a theoretical basis for developing PGPF-based green cultivation strategies for *S. divaricata* and to offer new microbial regulation approaches for the sustainable production of medicinal plants.

## 2. Results

### 2.1. Aspergillus neoalliaceus MR-86 Effects on Saposhnikovia divaricata Growth

Strain MR-86 formed distinct phosphate-solubilizing halos on inorganic phosphorus solid medium (Supplementary Figure S1A), indicating its ability to solubilize inorganic phosphate. Further quantification of phosphorus solubilization revealed that when calcium phosphate was supplied as the phosphorus source, the soluble phosphorus concentration produced by MR-86 during 9 days of fermentation initially increased and then gradually stabilized (Supplementary Figure S2A). Soluble phosphorus peaked at 667.90 mg/L on day 7, which was significantly higher than the values measured on days 1, 3, and 5 (*p* < 0.05). The pH of the fermentation broth declined progressively, reaching a minimum of 3.6 on day 9. These results demonstrate that strain MR-86 exhibits strong solubilizing activity toward calcium phosphate.

Similarly, when iron phosphate, aluminum phosphate, magnesium phosphate, zinc phosphate, and fluorapatite were provided as phosphorus sources, the amount of inorganic phosphorus solubilized by strain MR-86 gradually increased. Soluble phosphorus concentrations peaked on day 7 for iron, aluminum, and magnesium phosphates, reaching 185.36, 219.78, and 185.36 mg/L, respectively. In contrast, soluble phosphorus levels peaked on day 5 for zinc phosphate and fluorapatite at 528.70 mg/L and 131.73 mg/L, respectively. The pH of the fermentation broth progressively decreased over time, reaching its minimum on day 9, with values of 3.7, 3.67, 5.47, 4.32, and 4.52, respectively (Supplementary Figure S2B–F).

In addition to phosphate solubilization, MR-86 displayed multiple plant growth-promoting traits. When inoculated onto Ashby’s and potassium feldspar media, MR-86 grew successfully on nitrogen-free medium and formed clear halos on potassium feldspar medium, indicating its potential to utilize other nitrogen sources and K-releasing capacities (Supplementary Figure S1B,C). As shown in Supplementary Figure S1D, colorimetric assays confirmed that MR-86 secreted indole-3-acetic acid (IAA), with an IAA concentration of 18.23 μg/mL in the fermentation supernatant (Supplementary Table S3). On CAS agar plates (Supplementary Figure S1E), orange-yellow or transparent halos developed around MR-86 colonies, revealing its ability to produce siderophores, with a siderophore synthesis rate of 92.07% (Supplementary Table S3).

### 2.2. PCR Amplification Detection of Aflatoxin Biosynthesis Genes in Aspergillus neoalliaceus MR-86

As shown in Supplementary Figure S3, PCR amplification assays did not detect the presence of the *aflR*, *aflS*, or *omtA* genes.

### 2.3. Effects of Aspergillus neoalliaceus MR-86 on the Growth of Saposhnikovia divaricata

**Figure 1:**
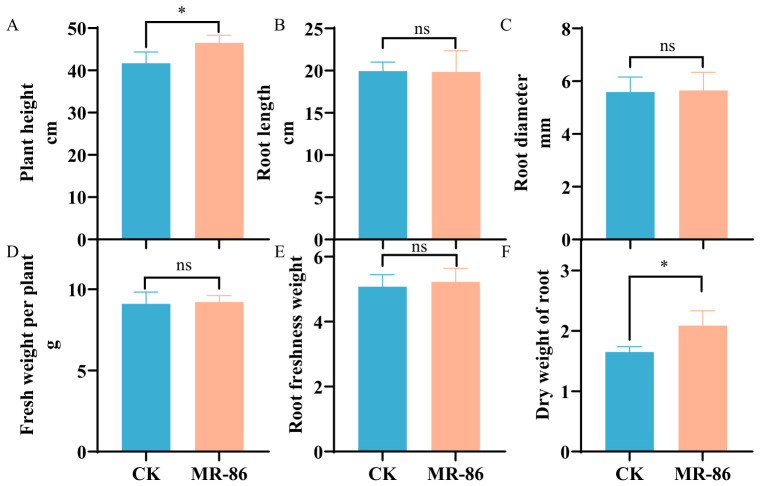
Effect of *Aspergillus neoalliaceus* MR-86 Inoculation on growth indicators of *Saposhnikovia divaricata*. (**A**): Plant height. (**B**): Root length. (**C**): Root diameter. (**D**): Fresh weight per plant. (**E**): Root freshness weight. (**F**): Dry weight of root. Data are presented as mean ± standard deviation (*n* = 5). * Indicates statistically significant differences between treatments (*p* < 0.05), as assessed by one-way analysis of variance (ANOVA) followed by Fisher’s least significant difference (LSD) post hoc test. ns indicates no significant difference (*p* ≥ 0.05).

Inoculation with MR-86 significantly promoted the growth of 2-year-old *S. divaricata* plants. As shown in Figure 1 and Supplementary Table S4, the plant height and root dry weight of *S. divaricata* inoculated with MR-86 reached 46.5 cm, a 10.32% increase compared with the water control (CK) (*p* < 0.05; Cohen’s d: 2.113, 95% CI: 0.566 to 3.66, Figure 1A), and the root dry weight of *S. divaricata* inoculated with MR-86 reached 2.09 g, a 21.05% increase compared with the water control (CK) (*p* < 0.05; Cohen’s d: 2.375, 95% CI: 0.756 to 3.994, Figure 1F).

### 2.4. Effects of Aspergillus neoalliaceus MR-86 on Rhizosphere Soil Chemical Properties and Enzyme Activities

**Figure 2:**
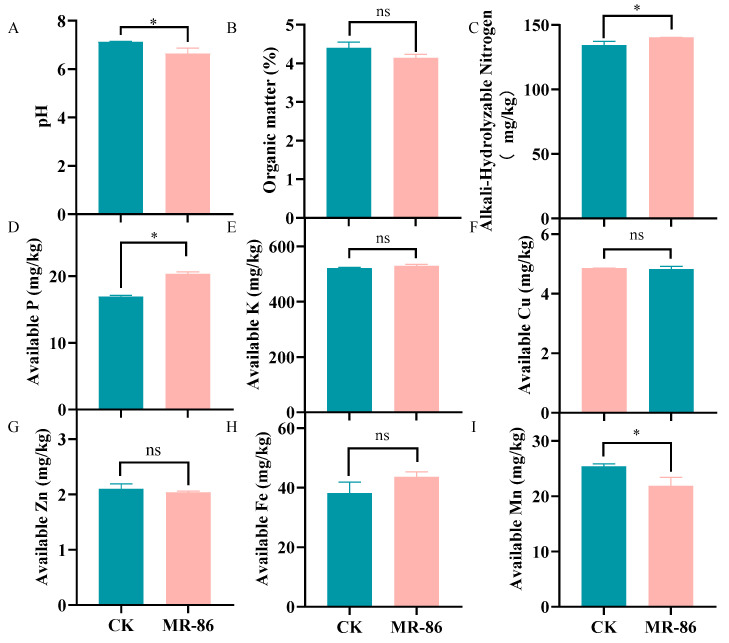
Effect of *Aspergillus neoalliaceus* MR-86 Inoculation on Rhizospheric Soil Nutrient Contents of *Saposhnikovia divaricata*. (**A**): pH. (**B**): Organic matter (SOM) content. (**C**): Alkaline-hydrolyzable nitrogen (AHN) content. (**D**): Available phosphorus (AP) content. (**E**): Available potassium (AK) content. (**F**): Available copper (ACu) content. (**G**): Available zinc (AZn) content. (**H**): Available iron (AFe) content. (**I**): Available manganese (AMn) content. Data are presented as mean ± standard deviation (*n* = 5). * Indicates statistically significant differences between treatments (*p* < 0.05), as assessed by one-way analysis of variance (ANOVA) followed by Fisher’s least significant difference (LSD) post hoc test. ns indicates no significant difference (*p* ≥ 0.05).

To investigate whether strain MR86 exerts its growth-promoting effects by modifying the rhizosphere environment, we further analyzed changes in the chemical properties and enzyme activities of the rhizosphere soil of *S. divaricata*. Inoculation with MR-86 significantly altered soil chemical properties (Figure 2 and Supplementary Table S5). In the MR-86 treatment, soil pH was 6.64, representing a 6.74% reduction compared with the control (CK, *p* < 0.05, Cohen’s d: 3.425, 95% CI: 0.912 to 5.938; Figure 2A). The soil alkaline-hydrolyzable nitrogen (AHN) contents reached 140.40 mg/kg in the MR-86 treatment, which was a 4.22% increase compared to CK (*p* < 0.05, Cohen’s d: 4.238, 95% CI: 1.355 to 7.121; Figure 2C), and the soil available phosphorus (AP) contents reached 20.30 mg/kg in the MR-86 treatment, which was a 16.6% increase compared to CK, respectively (*p* < 0.05, Cohen’s d: 12.867, 95% CI: 5.413 to 20.321; Figure 2D). Additionally, MR-86 treatment significantly reduced available manganese (AMn) content by 13.80% compared to CK (*p* < 0.05, Cohen’s d: −3.071, 95% CI: −5.433 to −0.709; Figure 2F).

**Figure 3:**
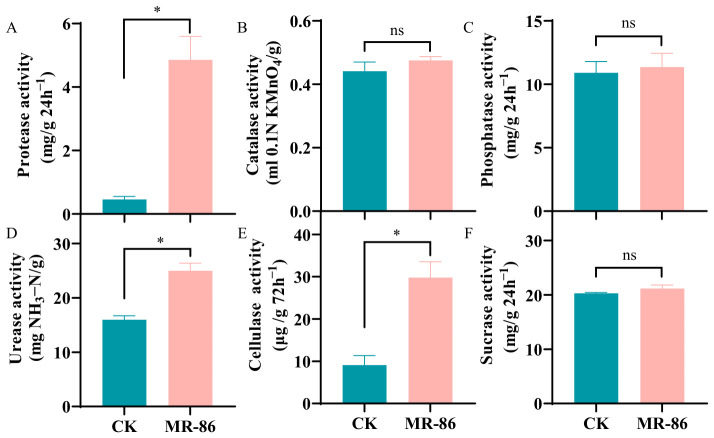
Effect of *Aspergillus neoalliaceus* MR-86 Inoculation on Rhizospheric Soil Enzyme Activity of *Saposhnikovia divaricata*. (**A**): Protease activity. (**B**): Catalase activity. (**C**): Phosphatase activity. (**D**): Urease activity. (**E**): Cellulase activity. (**F**): Sucrase activity. Data are presented as mean ± standard deviation (*n* = 5). * Indicates statistically significant differences between treatments (*p* < 0.05), as assessed by one-way analysis of variance (ANOVA) followed by Fisher’s least significant difference (LSD) post hoc test. ns indicates no significant difference (*p* ≥ 0.05).

Inoculation with MR-86 significantly enhanced the activities of soil protease activity (S-NPT) reaching 0.97 mg/g, representing an increase of 90.54% compared with CK (*p* < 0.05, Cohen’s d: 8.311, 95% CI: 3.344 to 13.278; Figure 3A and Supplementary Table S5). The activities of soil urease activity (S-UE) reached 25.00 mg/g in the MR-86 treatment, representing an increase of 36.05% compared with CK (*p* < 0.05, Cohen’s d: 8.111, 95% CI: 3.251 to 12.971; Figure 3D and Supplementary Table S5). The activities of soil cellulase activity (S-CL) reached 29.80 μg/g, representing an increase of 69.50% compared with CK (*p* < 0.05, Cohen’s d: 6.688, 95% CI: 2.579 to 10.79; Figure 3E and Supplementary Table S5). These results indicate that MR-86 can markedly increase nutrient availability and boost enzyme activities in rhizosphere soil, suggesting that it optimizes the rhizosphere nutrient environment by promoting nutrient supply and transformation processes.

### 2.5. Effects of Aspergillus neoalliaceus MR-86 on the Rhizosphere Microbial Community, Structure, and Diversity of Saposhnikovia divaricata

#### 2.5.1. Effects of *Aspergillus neoalliaceus* MR-86 on Rhizosphere Soil Microbial Diversity

Changes in soil chemical properties and enzyme activities are often closely associated with microbial community diversity. To further explore whether MR-86 indirectly promotes the growth of *S. divaricata* by regulating rhizosphere microecology, we analyzed shifts in rhizosphere microbial community structure. As shown in Supplementary Table S6, 8,510,230 valid bacterial (16S rRNA) sequences and 8,369,120 valid fungal (ITS) sequences were obtained from rhizosphere soil samples. After paired-end assembly, low-quality reads were filtered, and chimeric sequences were removed from the raw sequencing data. A total of 37,404 bacterial ASVs and 7048 fungal ASVs were identified across all rhizosphere soil samples.

Alpha diversity analysis based on high-throughput sequencing (Supplementary Figure S4) revealed that inoculation with MR-86 had no significant effect on the Shannon and Chao1 indices of the rhizosphere bacterial community (Supplementary Figure S4A,B), indicating that overall bacterial diversity remained stable. In contrast, MR-86 treatment significantly reduced the Shannon index of fungal communities (Supplementary Figure S4C). The fungal Chao1 index reached its maximum in the MR-86 treatment, but no significant differences were observed among treatments (Supplementary Figure S4D).

Principal coordinate analysis (PCoA) further revealed overall differences in microbial community structure between the two treatments. For the bacterial community, the first and second axes explained 15.2% and 13.7% of the total variation, respectively (Supplementary Figure S4E). For the fungal community, the two axes accounted for 29.9% and 12.7% of the variation, respectively (Supplementary Figure S4F). PERMANOVA confirmed significant differences between the two treatments (*p* < 0.05), demonstrating that MR-86 inoculation altered the microbial community structure in the rhizosphere soil of *S. divaricata*.

#### 2.5.2. Analysis of Changes and Differences in Bacterial and Fungal Community Composition

**Figure 4:**
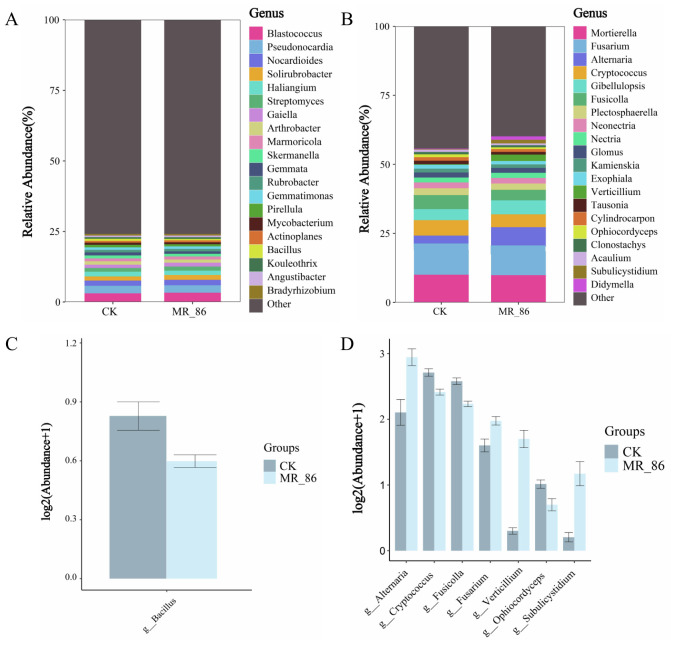
Comparative analysis of the relative abundance differences in rhizosphere soil bacteria and fungi after MR-86 inoculation. (**A**): Top 20 bacterial and fungal species compositions in the MR-86 inoculation group at the genus (**A**) and genus (**B**) levels. (**C**): Comparison of differences in bacterial genus-level relative abundance between the CK and MR-86 treatments. (**D**): Comparison of differences in fungal genus-level relative abundance between the CK and MR-86 treatments. All comparisons were performed using the Wilcoxon rank-sum test (Wilcox test; *p* < 0.05), Benjamini–Hochberg FDR < 0.1.

To investigate the effects of MR-86 inoculation on the rhizosphere bacterial community of *S. divaricata* at the phylum level (Supplementary Figure S5A), the five most abundant bacterial phyla were Actinomycetota (average relative abundance: 38.71%), Pseudomonadota (average relative abundance: 13.49%), Acidobacteriota (average relative abundance: 10.93%), Chloroflexota (average relative abundance: 11.37%), and Planctomycetota (average relative abundance: 9.73%), accounting for more than 84.23% of the total bacterial relative abundance. As shown in Figure 4A, the dominant bacterial genera were mainly *Blastococcus* and *Pseudonocardia*, each with a relative abundance of more than 2%. Among them, MR-86 treatment reduced *Bacillus* abundance compared to CK (Figure 4C).

Regarding the influence of MR-86 inoculation on the rhizosphere fungal community (Supplementary Figure S5B), the dominant fungal phyla mainly included Ascomycota, Basidiomycota, Entomophthoromycota, Glomeromycota, and Mucoromycota, representing more than 90% of the total fungal relative abundance. As shown in Figure 4B, the dominant fungal genera were primarily *Mortierella*, *Fusarium*, *Alternaria*, *Cryptococcus*, *Gibellulopsis*, *Fusicolla*, *Plectosphaerella*, and *Neonectria*, each with a relative abundance of more than 2%. Among them, MR-86 treatment increased the relative abundances of *Alternaria*, *Fusarium*, *Verticillium*, and *Subulicystidium*, while reducing those of *Cryptococcus*, *Fusicolla*, and *Ophiocordyceps* (Figure 4D).

**Figure 5:**
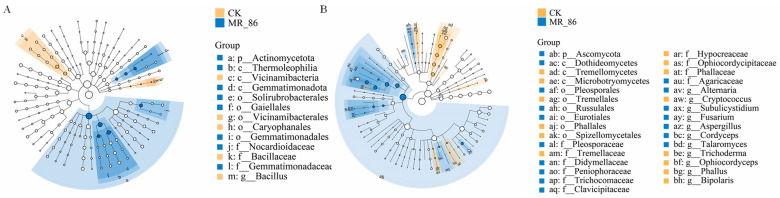
Comparative analysis of LEfSe differences between bacteria (**A**) and fungi (**B**). Linear discriminant analysis (LDA > 3.0 and *p* < 0.05). The concentric circles radiating out from the center in the figure represent the six classification levels from kingdom to genus. Each node represents the species classification at that level, and the node size is proportional to the species’ abundance.

Linear discriminant analysis effect size (LEfSe) was used to identify differentially abundant microbial taxa in the rhizosphere soil of *S. divaricata* under different treatments (Figure 5). A total of 13 significantly enriched bacterial taxa and 31 fungal taxa were identified. Inoculation with MR-86 led to significant enrichment of both bacterial and fungal communities compared with CK. At the phylum level, MR-86 treatment specifically enriched Actinomycetota and Ascomycota. At the genus level, MR-86 specifically enriched six fungal genera (*Alternaria*, *Subulicystidium*, *Fusarium*, *Aspergillus*, *Cordyceps*, and *Talaromyces*). These differential biomarker taxa likely play key roles in rhizosphere microecological shifts mediated by strain MR-86.

#### 2.5.3. Microbial Co-Occurrence Network Analysis

**Figure 6:**
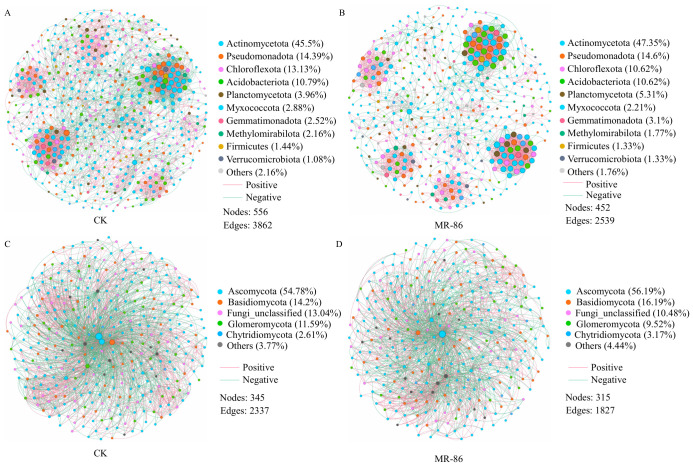
Co-occurrence network analysis of bacteria and fungi. (**A**,**C**): CK group. (**B**,**D**): MR-86 group. The pink lines indicate the co-occurrence relationship between two connected nodes, while the green lines represent the mutually exclusive relationship. Different nodes are distinguished by color to represent different categories, and node size indicates the strength of their connections. RMT: |r| > 0.82, SparCC: *p* < 0.05.

Based on sequencing results across different treatments, bacterial and fungal cross-domain co-occurrence networks were constructed. As shown in Figure 6A,B, the bacterial co-occurrence networks consisted of 556 and 452 nodes in the CK and MR-86 treatments, respectively. In both networks, nodes were mainly affiliated with Actinomycetota (CK: 45.5%, MR-86: 47.35%), Pseudomonadota (CK: 14.39%, MR-86: 14.6%), Chloroflexota (CK: 13.13%, MR-86: 10.62%), Acidobacteriota (CK: 10.79%, MR-86: 10.62%), Planctomycetota (CK: 3.96%, MR-86: 5.31%), Myxococcota (CK: 2.88%, MR-86: 2.21%), Gemmatimonadota (CK: 2.52%, MR-86: 3.1%), Methylomirabilota (CK: 2.16%, MR-86: 1.77%), Firmicutes (CK: 1.44%, MR-86: 1.33%), and Verrucomicrobiota (CK: 1.08%, MR-86: 1.33%). The networks comprised 3862 and 2539 edges, respectively. As shown in Supplementary Figure S6, CK treatment showed significantly higher node number, edge number, average degree, average path length, betweenness centralization, number of negative edges, and network diameter than MR-86, whereas MR-86 had significantly more positive edges and a higher clustering coefficient. Robustness analysis based on natural connectivity showed that the MR-86 bacterial network maintained higher connectivity than the CK network during sequential node removal in both weighted and unweighted networks (Supplementary Figure S8A), indicating enhanced network robustness and resistance to perturbation.

As shown in Figure 6C,D, the fungal co-occurrence networks consisted of 345 and 315 nodes in the CK and MR-86 treatments, respectively. In both networks, nodes were mainly affiliated with Ascomycota (CK: 54.78%, MR-86: 56.19%), Basidiomycota (CK: 14.2%, MR-86: 16.19%), Fungi_Unclassified (CK: 13.04%, MR-86: 10.48%), Glomeromycota (CK: 11.59%, MR-86: 9.52%), and Chytridiomycota (CK: 2.61%, MR-86: 3.17%). The networks comprised 2337 and 1827 edges, respectively. As shown in Supplementary Figure S7, CK treatment was associated with significantly higher node number, average path length, clustering coefficient, modularity, number of positive edges, and network diameter, whereas MR-86 treatment was associated with significantly higher network density, degree centralization, and number of negative edges. In contrast to the bacterial network, the fungal network under MR-86 treatment exhibited lower natural connectivity during sequential node removal in both weighted and unweighted networks (Supplementary Figure S8B), indicating reduced robustness and greater sensitivity to perturbation.

### 2.6. Correlation Analysis Between Microbial Taxa and Soil Environmental Factors

**Figure 7:**
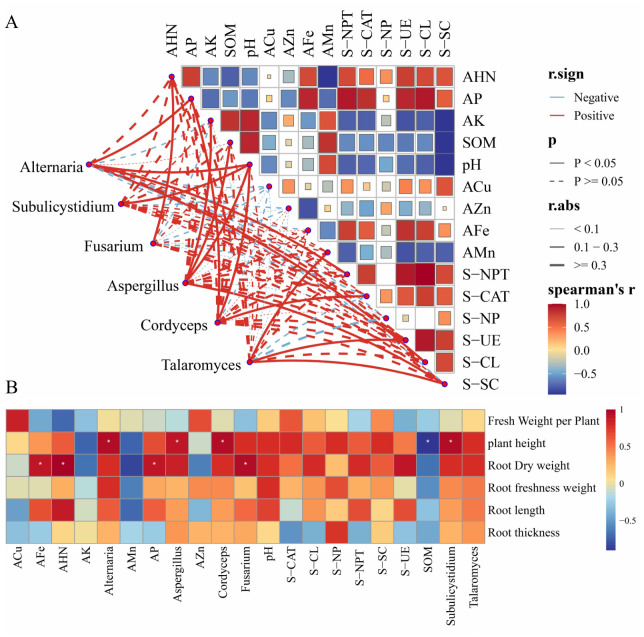
Correlation analysis of soil environmental factors, key microbial groups, and *Saposhnikovia divaricata* growth indicators. (**A**): Mental analysis of rhizosphere soil microorganisms and environmental factors of *Saposhnikovia divaricata*. The red lines indicate a positive correlation between the two connected nodes, while the blue lines indicate a negative correlation. The dotted lines indicate *p* ≥ 0.05, and the solid lines indicate *p* < 0.05. (**B**): Correlation analysis between growth indicators, soil factors, and microbial communities. It is characterized by the Pearson correlation coefficient. The red and blue blocks represent positive and negative correlations, respectively. The asterisks (*) in the blocks indicate statistical significance: * *p* < 0.05. All correlation analyses were performed using Spearman’s rank correlation coefficient.

Mantel test analysis revealed remarkable correlations between key microbial genera and soil physicochemical indices (Figure 7A). *Alternaria* was significantly positively associated with soil AP, pH, S-NPT, and S-CL (*p* < 0.05). *Subulicystidium* showed a positive correlation with soil pH (*p* < 0.05). *Fusarium* was positively correlated with AHN (*p* < 0.05). *Aspergillus* displayed significantly positive relationships with AK, SOM, pH, and S-SC (*p* < 0.05). *Cordyceps* was positively correlated with AP, pH, and S-CAT (*p* < 0.05), while *Talaromyces* was positively linked to soil AMn, S-UE, and S-SC (*p* < 0.05).

### 2.7. Relationships Between Growth Traits, Soil Properties, and Microbial Communities

In summary, the application of strain MR-86 notably altered the physicochemical properties, enzymatic activities, and composition of microbial communities within the rhizosphere soil. An analysis using Spearman correlation was performed to uncover the relationships between the observed soil changes and the growth parameters of *S. divaricata*, with the findings illustrated in Figure 7B. The plant height showed a significant positive relationship with the *Subulicystidium*, *Aspergillus*, *Cordyceps*, and *Alternaria* (*p* < 0.05) but had a significant negative relationship with soil SOM (*p* < 0.05). Additionally, root dry weight was significantly and positively connected with soil AHN, AFe, AP, and *Fusarium*.

### 2.8. Rhizosphere Microbial Functional Prediction

The functional prediction of bacterial communities in the two treatments was performed using PICRUSt2, which is based on the 16S rRNA gene database. Sequencing data were mapped to the KEGG functional database to obtain the corresponding functional profiles and abundance for each sample. At the Level 3 functional hierarchy, 298 functional subcategories were annotated, and the top 20 pathways by relative abundance are shown in Supplementary Figure S9A. Following MR-86 inoculation, pathways including ether lipid metabolism, glycosphingolipid biosynthesis–ganglio series, electron transfer carriers, flavone and flavonol biosynthesis, biosynthesis of type II polyketide backbone, biosynthesis of 12-, 14- and 16-membered macrolides, inositol phosphate metabolism, and bacterial toxins were predicted to be enriched in MR-86 treatment.

Functional prediction of the rhizosphere fungal communities was further performed using FUNGuild based on trophic modes. In this study, only Probable/Highly Probable guilds were retained, while low-confidence categories were excluded (Supplementary Figure S9B and Supplementary Table S7). According to the guilds’ classification, the functional guilds include plant pathogen, animal pathogen, wood saprotroph, soil saprotroph, litter saprotroph, fungal parasite, arbuscular mycorrhizal, dung saprotroph, ectomycorrhizal, and endomycorrhizal. MR-86 inoculation was predicted to increase the relative abundances of several functional guilds, including ectomycorrhizal and wood saprotrophic guilds, compared with CK.

## 3. Discussion

Microorganisms are a vital component of soil ecosystems and drive key ecological processes, including nutrient cycling and organic matter decomposition [27]. Accordingly, regulating the composition and function of soil microbial communities is critical for sustainable agricultural development. Among beneficial microorganisms, plant growth-promoting rhizobacteria (PGPR) can improve soil nutrient availability, stimulate crop growth, and maintain the ecological balance of soil microbial communities [28,29]. In recent years, PGPF/PGPR have been widely applied to various crops, including rice, maize, watermelon, and strawberry [30,31,32,33], yet systematic studies on their effects on medicinal plants such as *S. divaricata* remain limited [34,35,36,37]. Our results revealed that strain MR-86 exhibited phosphate-solubilizing, nitrogen-free medium growth, and potassium-releasing, IAA-producing, and siderophore-secreting capabilities, confirming its status as a functional plant growth-promoting microorganism. In addition, PCR amplification assays did not detect the presence of the aflatoxin biosynthesis-related genes *aflR*, *aflS*, and *omtA* in strain MR-86, suggesting that this strain may lack key genetic components associated with aflatoxin production. Pot experiments further validated its growth-promoting efficacy: inoculation with MR-86 significantly increased the plant height and root dry weight of *S. divaricata* by 10.32% and 21.05%, respectively. These findings align with the growth-promoting effects of plant growth-promoting fungi (PGPF) observed in diverse crops [38], further supporting the potential of MR-86 as a growth-promoting agent in medicinal plants.

As a fundamental soil property, nutrient availability plays a pivotal role in determining medicinal plant yield formation. Adequate nutrient availability can regulate rhizosphere microbial activity and nutrient cycling, thereby promoting root development, nutrient uptake, and biomass accumulation, thereby enhancing plant growth and yield. Our results demonstrated that application of the rhizosphere growth-promoting fungus MR-86 reduced soil pH and significantly increased the contents of available nitrogen and available phosphorus, indicating enhanced soil nutrient supply capacity. Soil enzymes serve as critical indicators reflecting soil nutrient transformation [39,40]. In this study, MR-86 inoculation significantly elevated the activities of soil protease, urease, and cellulase. Protease and urease are key catalysts involved in soil nitrogen transformation, and their enhanced activities directly regulate nitrogen conversion and soil nitrogen-supplying capacity [41], suggesting that MR-86 may accelerate the mineralization of soil organic nitrogen, thereby providing more available nitrogen for plant uptake. Meanwhile, the increased cellulase activity implies that MR-86 may promote the decomposition and turnover of soil organic matter. Collectively, MR-86 likely sustains *S. divaricata* growth by enhancing soil nutrient availability and facilitating nutrient transformations.

High-throughput sequencing results showed that inoculation with MR-86 altered the rhizosphere soil microbial community structure of *S. divaricata* but had a minor effect on bacterial α-diversity. Moreover, MR-86 treatment enriched microbial taxa, including *Alternaria*, *Subulicystidium*, *Fusarium*, *Aspergillus*, *Cordyceps*, and *Talaromyces*, which may play important roles in organic matter decomposition, nutrient cycling, or plant–microbe interactions. To further elucidate microbial interactions, co-occurrence networks were constructed, and network topological features were analyzed to assess community complexity and the intensity of interspecies interactions [42]. In the bacterial networks, the dominant bacterial phyla were similar between the CK and MR-86 treatments. Network topology analysis showed that MR-86 inoculation altered bacterial interaction patterns in the rhizosphere, with increased positive interactions and clustering characteristics that may promote closer microbial associations. In addition, the increased clustering coefficient and positive interactions observed in the MR-86 bacterial network may contribute to its enhanced robustness and resistance to perturbation. In the fungal co-occurrence networks, Ascomycota and Basidiomycota remained the dominant phyla, with no noticeable shifts in phylum composition. Topological analysis indicated that MR-86 treatment altered fungal interaction patterns, with increased network centralization, shorter propagation paths, and a higher proportion of negative interactions, suggesting intensified competitive relationships among fungal taxa. In addition, the decreased robustness of the fungal network under MR-86 treatment may be associated with increased network centralization and stronger competitive relationships, making the network more sensitive to perturbation.

To clarify the intrinsic relationships between specific microbial taxa and soil environmental variables, Mantel tests revealed significant positive correlations between multiple microbial taxa enriched under MR-86 treatment and soil nutrient indices and enzyme activities. Notably, *Talaromyces* has been shown to possess plant growth-promoting traits that enhance wheat growth and the efficiency of soil nutrient utilization [43]. We infer that the microbial taxa enriched by MR-86 inoculation may be associated with nutrient availability in the rhizosphere. Spearman’s correlation analysis further indicated that the enrichment of key microbial taxa under MR-86 treatment was positively associated with *S. divaricata* biomass. Therefore, further isolation, cultivation, and functional validation experiments are needed to determine whether these microbial taxa are regulated by MR-86 and whether they contribute to the growth-promoting effects on *S. divaricata*.

To explore the metabolic functional profiles of rhizosphere microbial communities following MR-86 inoculation, we performed KEGG functional prediction of bacterial communities using PICRUSt2 and ecological functional classification of fungal communities using FUNGuild, based on 16S rRNA and ITS gene sequencing data. Results showed that MR-86 treatment was predicted to enrich pathways related to flavonoid biosynthesis, bacterial toxins, and glycosphingolipid biosynthesis within the bacterial community. Enrichment of flavonoid biosynthesis-related pathways may reflect enhanced secondary metabolic activity in the rhizosphere microbiome, thereby strengthening plant–microbe interactions. Functional prediction of soil fungi via FUNGuild revealed that the relative abundance of functional guilds, including ectomycorrhizal and wood saprotrophs, was predicted to be enriched under MR-86 treatment. We speculate that strain MR-86 may accelerate the decomposition of soil organic matter and the release of nutrients by predicted enrichment of fungal saprotrophic metabolism [44,45], thus providing sustained nutritional support for the growth of *S. divaricata*.

## 4. Materials and Methods

### 4.1. Assessment of Plant Growth-Promoting Traits of Strain Aspergillus neoalliaceus MR-86

Our research group previously isolated a fungal strain from the rhizospheric soil of healthy *S. divaricate*, designated as No. MR-86, and deposited it at the Guangdong Microbial Culture Collection Center (GDMCC) with the accession number GDMCC No. 62083. The ITS, β-tubulin, and calmodulin sequences of *Aspergillus neoalliaceus* have been deposited in GenBank under accession numbers OK287151, PQ857578, and PQ857574 (These sequences were not generated as part of this study).

Strain MR-86 was cultured on National Botanical Research Institute’s Phosphate (NBRIP) agar medium at a temperature of 25 °C for a duration of 3 to 5 days [46]. During this period, the strain’s ability to solubilize phosphate was assessed by observing transparent halos of hydrolysis around the colonies, indicating successful phosphate solubilization. To quantify this solubilization process, a spore suspension of strain MR-86 was prepared and adjusted to a concentration of 1 × 10^7^ colony-forming units (CFU) per milliliter using sterile water. This spore suspension was then inoculated into NBRIP liquid medium at a concentration of 1% (*v*/*v*) and incubated at 25 °C, with continuous shaking at 170 revolutions per minute (rpm). The culture supernatants were collected at specific intervals—on days 1, 3, 5, 7, and 9—by centrifuging the cultures at 10,000 rpm for 10 min. To determine the soluble phosphorus content in the supernatants, the molybdenum–antimony colorimetric method was employed [47]. Additionally, the pH levels of the fermentation broth were meticulously monitored throughout the incubation period to provide further insight into the conditions affecting phosphate solubilization.

A standard curve was created utilizing phosphate standard solutions with specific concentrations of 0, 0.2, 0.4, 0.6, 0.8, and 1.0 mg/L. This procedure yielded a linear regression equation, y = 0.2909x − 0.0028, with a correlation coefficient R^2^ = 0.9996. Using this standard curve, the concentration of soluble phosphorus in the fungal supernatant was accurately determined. Furthermore, the phosphate-solubilizing capacity of the strain MR-86 was assessed by calculating the difference between the levels of soluble phosphorus in the fungal supernatant and those in the uninoculated control sample. To further investigate the solubilization capabilities of MR-86 with respect to various insoluble phosphates, tricalcium phosphate in the NBRIP medium was replaced with alternative phosphates, including iron phosphate, aluminum phosphate, magnesium phosphate, zinc phosphate, and fluorapatite. The resulting soluble phosphorus concentrations and pH levels were measured using the same previously established method. This comprehensive evaluation facilitates a deeper understanding of MR-86’s potential to solubilize different forms of phosphate, thereby enhancing its applicability in relevant biological and environmental contexts.

To evaluate the growth ability of the strain on nitrogen-free medium, it was inoculated onto Ashby’s nitrogen-free solid medium [48] and incubated at 25 °C for 5 days. The strain’s ability to grow on a nitrogen-free medium was used as an indicator of its capacity to survive without an external nitrogen source.

To assess the strain’s potassium-solubilizing capabilities, an assay was conducted using a potassium feldspar medium [49]. The strain was carefully inoculated into this specialized environment and incubated at 25 °C for 5 days. After this incubation period, a distinct, clear halo around the growth was observed. This halo formation is an important indication that the strain can effectively solubilize potassium.

The production of indole-3-acetic acid (IAA) by the strain MR-86 was investigated through the Salkowski colorimetric method [50]. MR-86 was inoculated into Potato Dextrose Broth (PDB) and allowed to ferment under controlled conditions for a period of seven days. Following this incubation period, the culture supernatant was collected for analysis of IAA content. To facilitate the detection of IAA, the supernatant was combined in equal parts with a color reagent containing FeCl_3_, known as the PC reagent [51]. This mixture was then placed in a dark environment for 30 min, allowing sufficient time for the color reaction to occur and confirming the production of IAA by the microorganism. Uninoculated PDB medium was used as the negative control, while a 50 μg/mL IAA standard solution mixed with an equivalent volume of PC reagent served as the positive control. A color similar to that of the standard indicated that the strain produced IAA [51]. To quantify IAA, standard solutions were prepared at concentrations of 0, 10, 20, 30, 40, 50, and 60 μg/mL, yielding a standard curve with the linear regression equation y = 0.0345x + 0.0135 (R^2^ = 0.9987). After color development, the optical density at 530 nm (OD_530_) of each sample was measured [52], and the concentration of IAA produced by MR-86 was calculated based on the standard curve.

Siderophore production assay: The siderophore-producing capacity of strain MR-86 was preliminarily evaluated employing the Chrome Azurol S (CAS) agar overlay assay [53]. The strain was inoculated onto CAS agar and incubated for 5 days. The formation of yellow or orange halos around the colonies was considered a preliminary indication of siderophore production. For quantitative analysis, a spore suspension of MR-86 (adjusted to 1 × 10^6^ CFU/mL with sterile water) was inoculated into PDB medium at a 5% (*v*/*v*) ratio, then incubated at 28 °C with shaking at 120 rpm for 7 days. The fermentation broth was centrifuged at 8000 rpm for 15 min to collect the supernatant, and uninoculated PDB medium served as the blank control. The supernatant was mixed with an equal volume of CAS assay solution and allowed to stand for 1 h, after which the absorbance at 630 nm (OD_630_, C) was measured. The absorbance of the blank, prepared by mixing uninoculated PDB medium with CAS solution (D), was used as a reference. The siderophore production rate (%) was calculated using the formula:

(1) $$\mathrm{Siderophore}\;\mathrm{production}\;\mathrm{rate}\;(\%)=\frac{(D-C)\times 100}{D}$$

*A. neoalliaceus* MR-86 was inoculated on Potato Dextrose Agar (PDA) plates and incubated in the dark at 25 °C for 7 days. The mycelia of MR-86 were then collected and ground into a fine powder using liquid nitrogen. According to the manufacturer’s instructions, MR-86 genomic DNA was extracted using the Fungi Genomic DNA Extraction Kit (D2300, Solarbio, Beijing, China). Primers *aflR*, *omtA*, and *aflS* were selected for amplification [54,55] as listed in Supplementary Table S1. PCR amplification conditions are provided in Supplementary Table S2. PCR products were detected by electrophoresis on 1% agarose gels.

### 4.2. Pot Experiment

The plant material used in this study was *S. divaricata* cultivated in the Medicinal Botanical Garden of Jilin Agricultural University (Changchun City, Jilin Province, China; 43°48′24″ N, 125°24′59″ E). All collected plant materials complied with national and local regulations and did not involve any protected species.

For inoculation experiments, *A. neoalliaceus* MR-86 was first cultured on PDA plates at 25 °C in the dark for 10 days. Spores were then harvested by gently rinsing the plates with sterile water, and the resulting suspension was filtered through three layers of sterile gauze to remove mycelial fragments. The spore suspension was adjusted to a concentration of 10^6^ CFU/mL and used for plant inoculation.

Uniform two-year-old *S. divaricata* plants were transplanted into polypropylene pots (bottom diameter 8.8 cm, top diameter 12 cm, height 32 cm), with one seedling per pot (during dormancy, the average root length is 15 cm, and the fresh root weight is 3.5 g). Growth promotion experiments were initiated one month after transplantation. The soil used was black soil with the following baseline properties: pH 6.91; alkaline-hydrolysable nitrogen (AHN) 137.76 mg/kg; soil organic matter (SOM) 4.55%; available phosphorus (AP) 18.89 mg/kg; available potassium (AK) 604.27 mg/kg; available copper (ACu) 4.78 mg/kg; available zinc (AZn) 2.07 mg/kg; available iron (AFe) 27.66 mg/kg; and available manganese (AMn) 19.41 mg/kg.

Two experimental groups were set up: treatment group A, inoculated with MR-86 (10^6^ CFU/mL), and control group CK, treated with distilled water. In the A treatment, each plant was irrigated at the root zone with 100 mL of the spore suspension, while the CK treatment received an equal volume of sterile water. Each treatment included five biological replicates, with ten pots per replicate, and all plants were cultivated under consistent natural field conditions. After two months of growth, rhizosphere soil and plant tissues were sampled separately from each group. Loosely bound soil was first removed by gentle shaking of the root systems, and tightly attached rhizosphere soil was subsequently collected with a brush [56,57]. For soil analyses, soil from all 10 pots in each replicate was thoroughly mixed to form a composite sample. Plant samples were immediately taken to the laboratory, rinsed clean, measured for relevant indicators, and then dried to constant weight in a 40 °C oven. Soil specimens were split into two aliquots: one portion was preserved at −80 °C for subsequent amplicon sequencing, while the other was air-dried at room temperature to determine soil physicochemical properties and enzyme activities.

### 4.3. Soil Chemical Analysis

Soil pH was determined via the potentiometric approach; soil SOM was quantified using the potassium dichromate oxidation method [58], while soil AHN was measured by the alkaline hydrolysis diffusion method [59]. Soil AP was analyzed through sodium bicarbonate extraction combined with UV-visible spectrophotometry [60], and soil AK was detected using ammonium acetate extraction coupled with flame photometry [61]. Soil ACu, AZn, AFe, and AMn were extracted with diethylenetriaminepentaacetic acid (DTPA) and further determined by inductively coupled plasma optical emission spectrometry (ICP-OES) [62].

### 4.4. Soil Enzyme Activities

The activity of soil sucrase (S-SC) was assessed using the colorimetric assay with 3,5-dinitrosalicylic acid [63]. Soil urease (S-UE) activity was measured by employing the indophenol blue colorimetric method [64]. The catalase (S-CAT) activity in the soil was evaluated through potassium permanganate titration [65]. Soil phosphatase (S-NP) activity was quantified using the disodium phenyl phosphate procedure [66]. Additionally, both soil protease (S-NPT) and cellulase (S-CL) activities were assessed using the TTC reduction assay [67]. The activities of these enzymes were reported as the amounts of glucose, NH_3_–N, H_2_O_2_, free phenol, and reduced TTC produced for every gram of dry soil after a 24-h incubation.

### 4.5. Extraction, Amplification, and Sequencing of Rhizosphere Soil DNA

Genomic DNA was extracted from soil samples utilizing a bead-beating DNA extraction kit. Bacterial DNA was extracted using the Bacterial Genomic DNA Extraction Kit (No. DP302-02, Tiangen, Beijing, China), while fungal DNA was extracted using the CTAB method. The integrity of the obtained DNA was assessed through 0.8% agarose gel electrophoresis, and its purity and concentration were subsequently evaluated. For bacterial community analysis, amplification of the V3–V4 hypervariable region of the 16S rRNA gene was performed using the primer pair 341F/805R [68], while the fungal ITS region was targeted with the ITS1FI2/ITS2 primers [69]. The evaluation of the amplicon libraries was performed with an Agilent 2100 Bioanalyzer (Agilent Technologies, Santa Clara, CA, USA), followed by high-throughput sequencing on the Illumina NovaSeq 6000 platform (LC Bio Technology Co., Ltd., Hangzhou, China). The initial raw sequencing reads were processed using the QIIME2 pipeline [49], which included sample demultiplexing and primer removal. Additional quality control measures, such as noise reduction, merging of paired-end reads, and chimera removal, were carried out using the DADA2 plugin. Amplicon sequence variants (ASVs) were resolved as exact sequence variants by DADA2. In addition to this identification process, an abundance table detailing the presence and quantity of each ASV was constructed, enabling a clearer understanding of the distribution of these variants within the analyzed samples. Furthermore, to accurately categorize the identified bacterial and fungal sequences, taxonomic classification was conducted. This classification involved aligning the sequences against the well-established SILVA database [50]. NT-16S was used as a reference database for 16S rRNA sequences, while ITS fungal sequences were annotated using the RDP database, with species assignment based on the UNITE database and a confidence threshold of 0.7.

### 4.6. Statistical Analyses

Statistical analyses were performed on indices of plant growth, soil chemical characteristics, and soil enzyme activities. To analyze the data, one-way ANOVA was utilized, followed by LSD post hoc tests for multiple comparisons. In addition, effect sizes were calculated using Cohen’s d, and 95% confidence intervals (CI) were reported to provide a standardized measure of the magnitude and uncertainty of differences between treatments [70]. The results were visualized using GraphPad Prism version 8.0.2.

To standardize comparisons, all samples were randomly subsampled (rarefied) to the minimum number of reads observed across all samples. Rarefied data were then used to calculate α-diversity (Chao1 and Shannon) and β-diversity metrics (Bray–Curtis distances). Differential abundance analyses of microbial taxa and functional predictions were conducted using the Wilcoxon rank-sum test (Wilcox test, *p* < 0.05) in combination with false discovery rate (FDR) correction (Benjamini–Hochberg, FDR < 0.1) [71]. To identify differentially abundant bacterial and fungal taxa among the treatments, linear discriminant analysis (LDA) in conjunction with LEfSe was employed, using screening criteria of LDA score > 3.0 and *p* < 0.05. In the co-occurrence network, ASVs with an abundance greater than 0.03% were selected as network nodes. Pairwise correlations between ASVs were calculated using the SparCC algorithm implemented in the SpiecEasi R package (v4.4.1), and only correlations with *p* < 0.05 were retained. The correlation threshold for the network was determined by Random Matrix Theory (RMT, |r| > 0.82) [72]. Network topological properties were calculated using igraph (R package, v4.4.1), and comparisons between MR-86 and CK networks were performed using *t*-tests. Separate co-occurrence networks were constructed for bacteria and fungi. This network was then visualized in Gephi (version 0.10.1) [73]. Key topological metrics for the network were computed, including the number of nodes, the number of edges, the average degree, the average path length, betweenness centralization, density, degree centralization, clustering coefficient, eigenvector centralization, modularity, the number of negative edges, the number of positive edges, and the network diameter. In addition, network robustness was evaluated based on natural connectivity during sequential node removal under both weighted and unweighted conditions. The functional prediction of bacterial communities was conducted using PICRUSt2 (2.2.0b) [74] with Nearest Sequenced Taxon Index (NSTI) < 0.15, while FUNGuild (v1.0) was utilized for the annotation of fungal trophic modes [75], retaining only probable/highly probable guild assignments and excluding low-confidence categories [76]. To examine the relationships between microbial communities and soil properties, Mantel tests were performed. Microbial community data were analyzed using a Bray–Curtis distance matrix, whereas soil physicochemical properties and enzyme activities were analyzed using a Euclidean distance matrix. Mantel tests were conducted with 999 permutations to assess statistical significance. Additionally, correlation heatmaps were generated using Spearman’s rank correlations to assess pairwise associations among microbial taxa, soil properties, and plant growth parameters. All correlation analyses were performed using the online analytical platform (https://www.omicstudio.cn/tool, accessed on 22 May 2026).

## 5. Conclusions

This study demonstrates that *A. neoalliaceus* MR-86 may influence the growth of *S. divaricata* by modulating soil health and the rhizosphere microecological environment. Our findings revealed that MR-86 inoculation not only enhanced soil nutrient availability and increased the activities of key enzymes (e.g., protease and urease) to accelerate soil nutrient transformation and organic matter decomposition, but also altered the rhizosphere microbial community structure and enriched multiple potential functional microbial taxa. While MR-86 inoculation did not alter the composition of dominant bacterial and fungal phyla, it significantly modified microbial interactions and the topology of microbial networks. This study preliminarily uncovers the growth-promoting effects of *A. neoalliaceus* MR-86 on *S. divaricata* and its regulatory potential on the rhizosphere microecology. From a microecological perspective, this research provides new insights and a theoretical basis for developing green cultivation technologies for medicinal plants.

## Data Availability

The original contributions presented in this study are included in the article. Further inquiries can be directed to the corresponding authors.

## Supplementary Materials

The following supporting information can be downloaded at: https://www.mdpi.com/article/10.3390/plants15111703/s1. Figure S1. Growth-promoting properties of strain MR-86; Figure S2. The ability of strain MR-86 to dissolve insoluble phosphorus; Figure S3 PCR amplification detection of aflatoxin and cyclopiazonic acid biosynthesis genes in *Aspergillus neoalliaceus* MR-86; Figure S4. α-diversity and β-diversity indices of rhizosphere microbial communities in different treatments; Figure S5. Top 10 bacterial (A) and top 9 fungal (B) species composition in the MR-86 inoculation group at the phylum levels; Figure S6. Comparisons of bacterial network topological properties between treatments were performed using t-tests; Figure S7. Comparisons of fungal network topological properties between treatments were performed using t-tests; Figure S8. Based on natural connectivity during sequential node removal under both weighted and unweighted conditions; Figure S9. Prediction of rhizosphere soil microbial metabolic function; Table S1. List of primer used for the detection of aflatoxin and cyclopiazonic acid biosynthesis genes; Table S2. PCR reaction conditions; Table S3. The content of IAA secreted by strain MR-86 and siderophores; Table S4. Cohen’s d and 95% confidence interval (CI) for plant growth parameters; Table S5. Cohen’s d and 95% confidence interval (CI) for soil chemical properties and enzyme activities; Table S6. Valid sequences and ASV numbers of bacteria and fungi were obtained from rhizosphere soil samples; Table S7. Probable and highly probable functional guilds.
