# Supplementary material for: Aspergillus neoalliaceus MR-86 Promotes the Growth of Saposhnikovia divaricata by Regulating the Rhizosphere Microbiome
Source: Plants (Basel). 2026 May 31;15(11):1703. doi: 10.3390/plants15111703 (PMC13258830; doi:10.3390/plants15111703)
Supplement: Supplementary file 1 [file plants-15-01703-s001.zip › plants-4310826-supplementary.pdf]

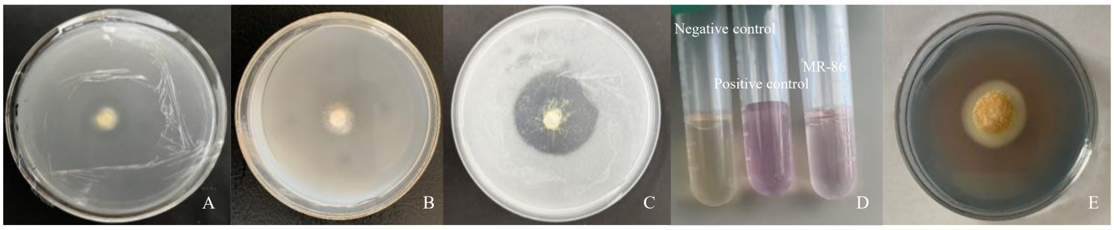

Figure S1. Growth-promoting properties of strain MR-86. A: Dissolved phosphorus. B: Ability to grow in a nitrogen-free medium. C: Potassium solubilization. D: Produced by IAA. E: Secretory siderophores.

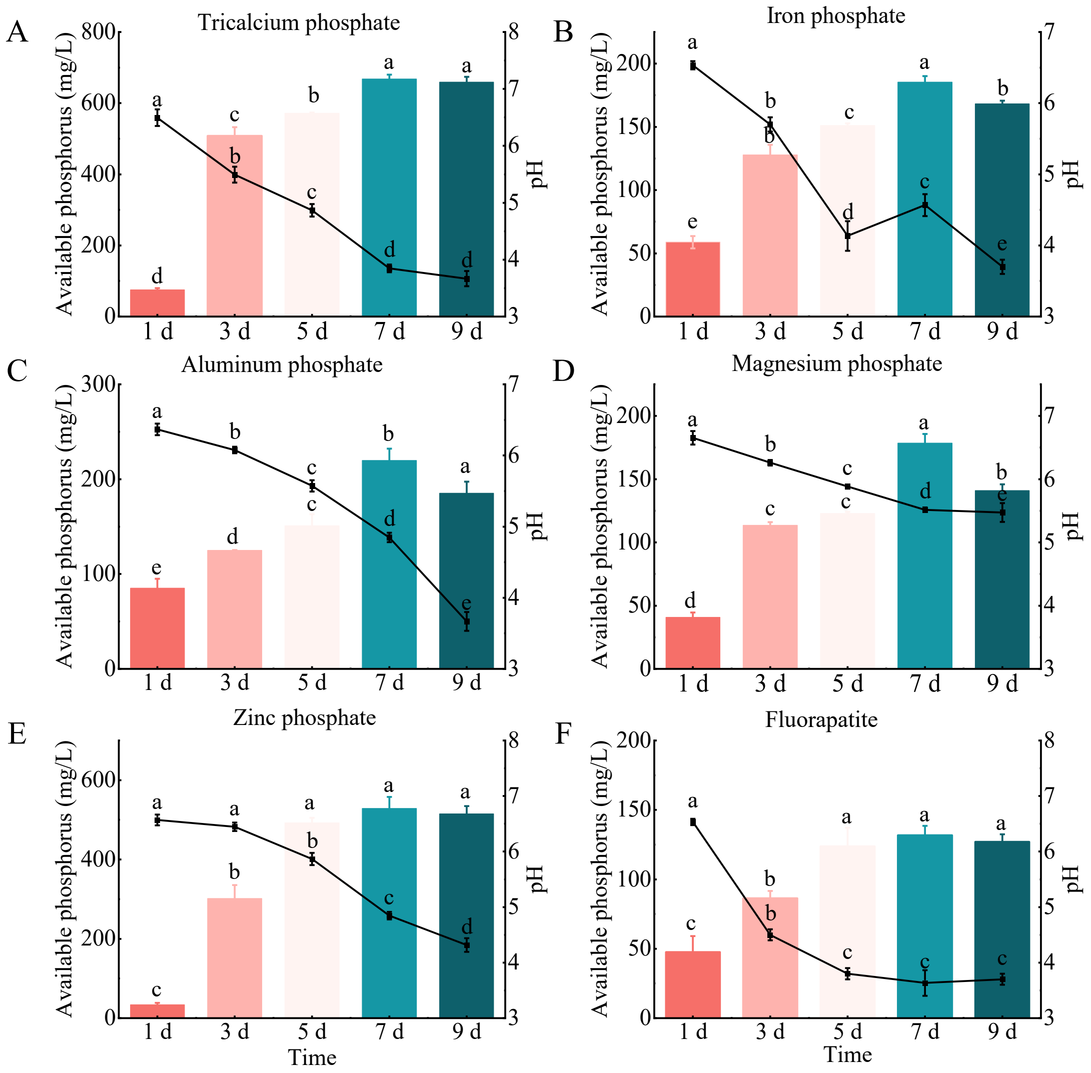

Figure S2. The ability of strain MR-86 to dissolve insoluble phosphorus. A: Solubility of tricalcium phosphate. B: Solubility of iron phosphate. C: Solubility of aluminum phosphate. D: Solubility of magnesium phosphate. E: Solubility of zinc phosphate. F: Solubility of fluorapatite. Data are presented as mean ± standard deviation (n = 3). Different lowercase letters denote statistically significant differences among treatments, as assessed by one-way analysis of variance (ANOVA) followed by Fisher's least significant difference (LSD) post hoc test ($p < 0.05$).

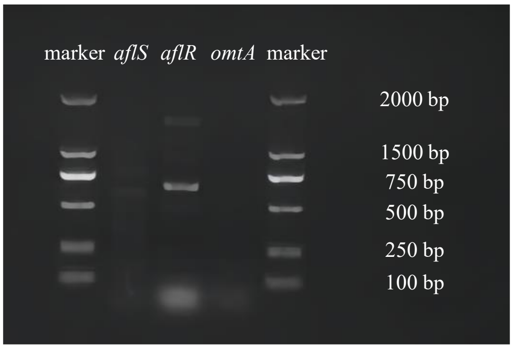

Figure S3 PCR amplification detection of aflatoxin and cyclopiazonic acid biosynthesis genes in *Aspergillus neoalliaceus* MR-86

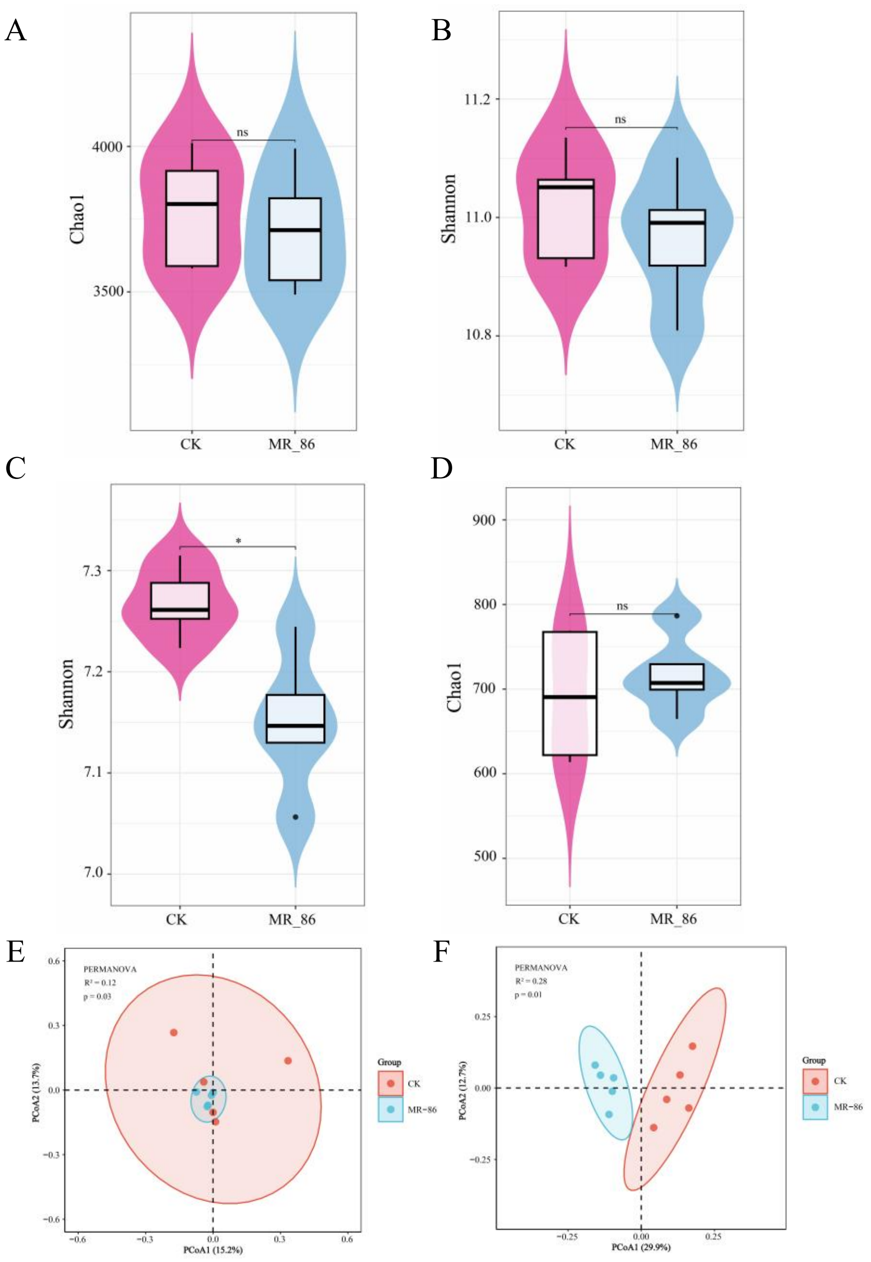

Figure S4. α-diversity and β-diversity indices of rhizosphere microbial communities in different treatments. A: Shannon index of bacteria. B: Chao1 index of bacteria. C: Shannon index of fungi. D: Chao1 index of fungi. E Principal coordinate analysis (PCoA) of soil bacterial communities. F: Principal coordinate analysis (PCoA) of soil fungal communities. * Wilcox test $p < 0.05$.

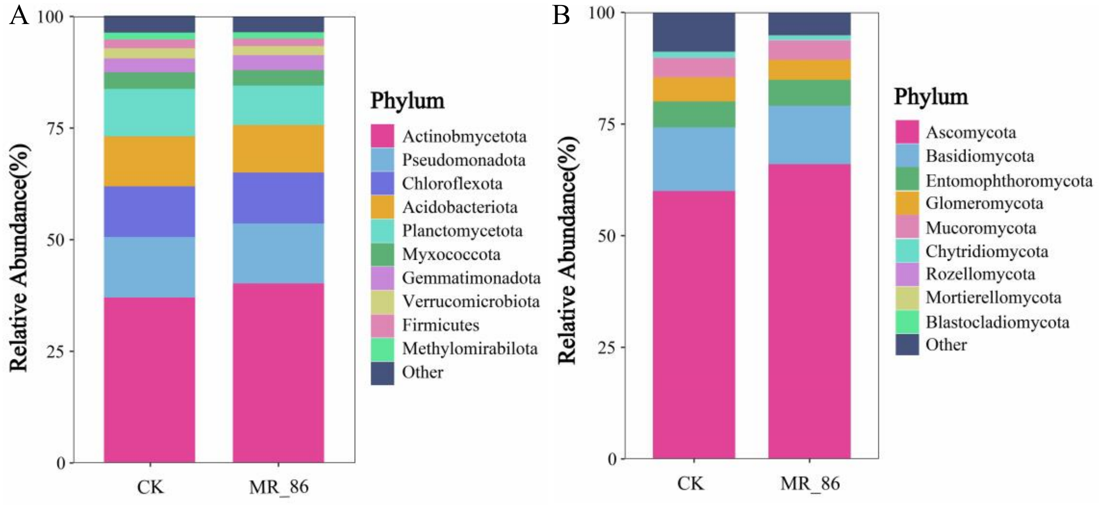

Figure S5. Top 10 bacterial (A) and top 9 fungal (B) species composition in the MR-86 inoculation group at the phylum levels.

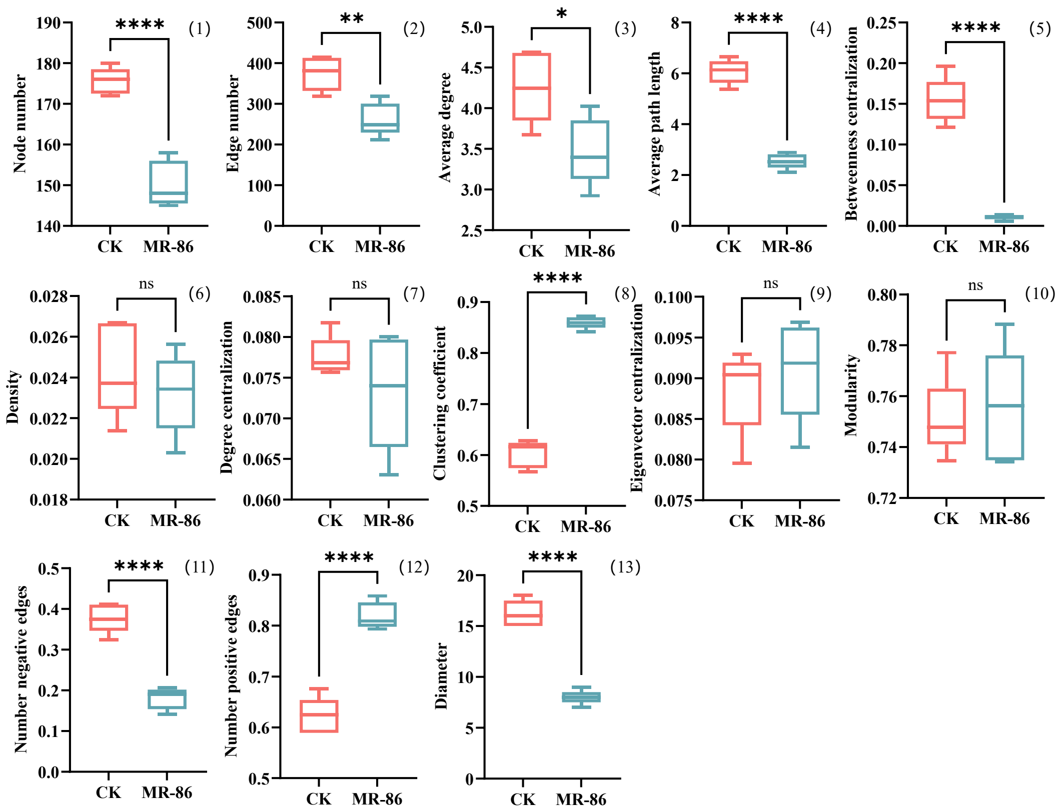

Figure S6. Comparisons of bacterial network topological properties between treatments were performed using t-tests. Significance levels are indicated as * $p < 0.05$, ** $p < 0.01$, *** $p < 0.005$, and **** $p < 0.001$. ns indicates no significant difference ($p \geq 0.05$).

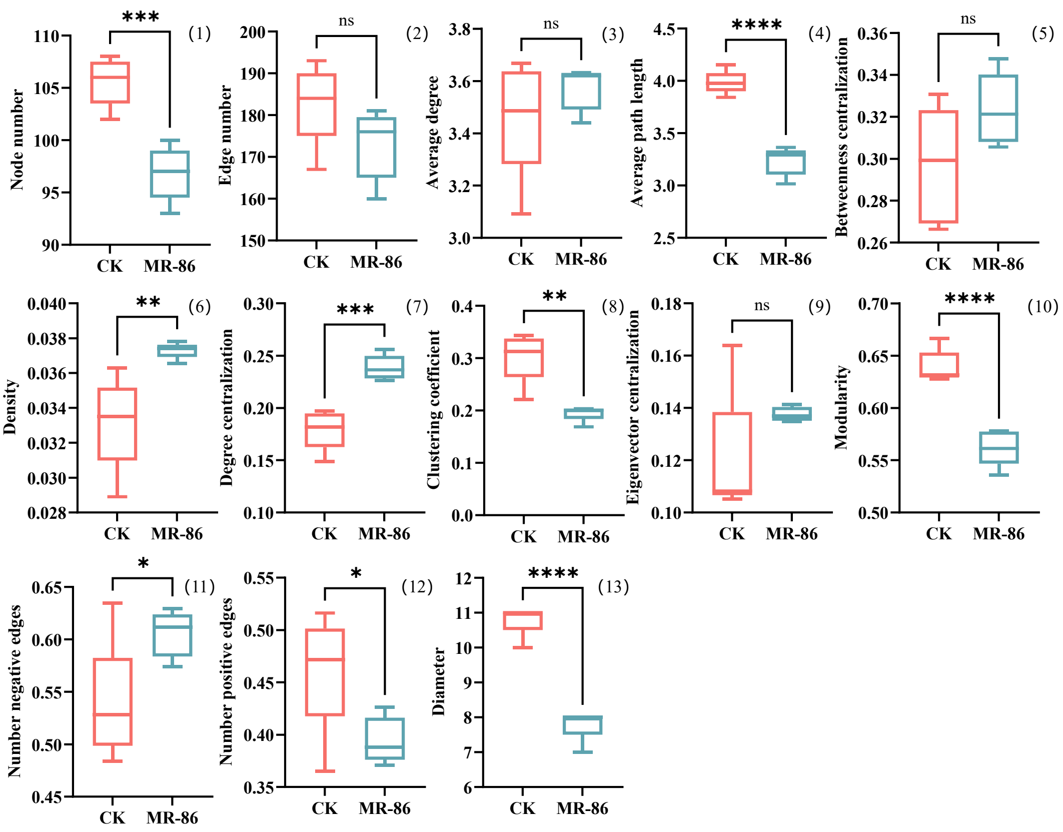

Figure S7. Comparisons of fungal network topological properties between treatments were performed using t-tests. Significance levels are indicated as * $p < 0.05$, ** $p < 0.01$, *** $p < 0.005$, and **** $p < 0.001$. ns indicates no significant difference ($p \geq 0.05$).

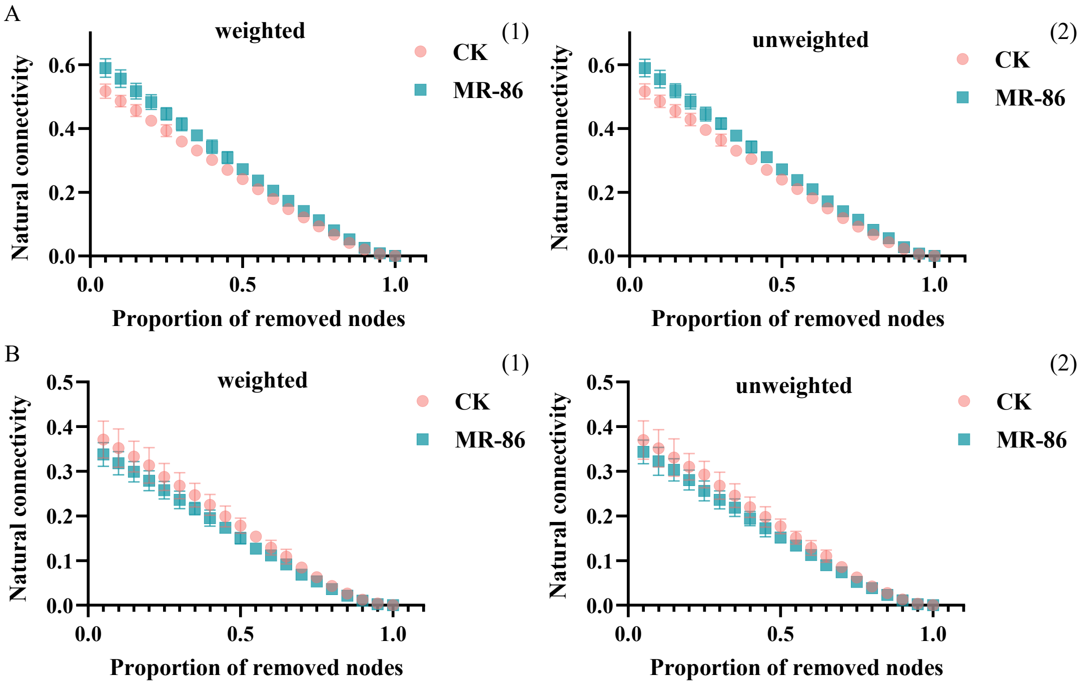

Figure S8. Based on natural connectivity during sequential node removal under both weighted and unweighted conditions. A: 16S RNA, B: ITS.

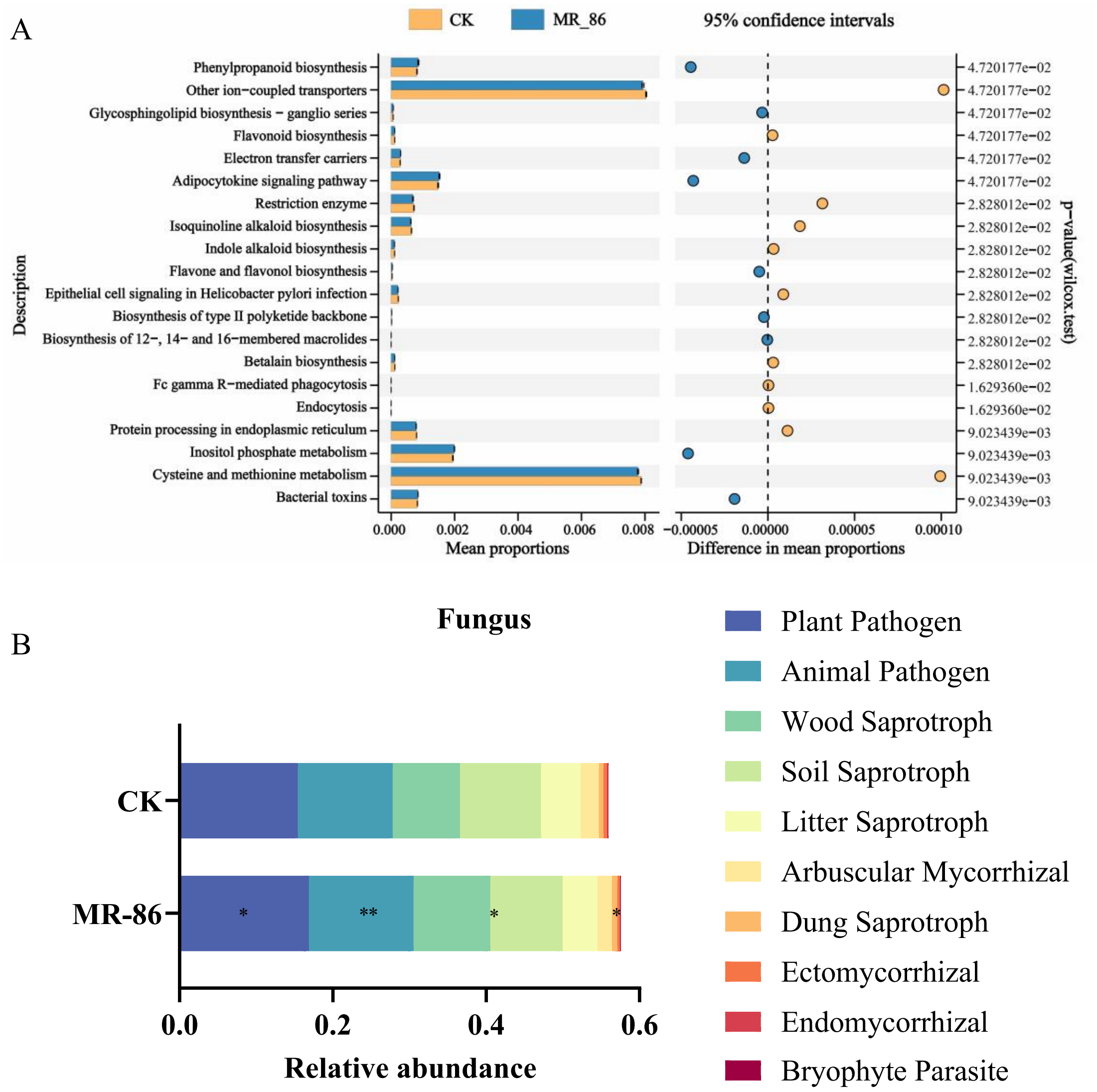

Figure S9. Prediction of rhizosphere soil microbial metabolic function. A: Differences of bacterial community function prediction in rhizosphere soil (Level 3 Functional Layer). B: Differences of fungal community function prediction in rhizosphere soil. All comparisons were performed using the non-parametric. Wilcox test with a significance level of *$p < 0.05$, ** $p < 0.01$, FDR < 0.1.

Table S1. List of primers used for the detection of aflatoxin and cyclopiazonic acid biosynthesis genes

Primers	Primers sequences
aflR	aflr1: 5′-TATCTCCCCCCGGGCATCTCCCGG-3′
	aflr2: 5′-CCGTCAGACAGCCACTGGACACGG-3′
omtA	omt1: 5′-GGCCCGGTTCCTTGGCTCCTAAGC-3′
	omt2: 5′-CGCCCCAGTGAGACCCTTCCTCG-3′
aflS	AflS-1for: 5′-TGAATCCGTACCCTTTGAGG-3′
	AflS-2rev: 5′-GGAATGGGATGGAGATGAGA-3′

Table S2. PCR reaction conditions

Primers	PCR Reaction Condition	Cycle	Size (bp)
omtA	Initial denaturation: 95 °C, 1 min<br>Denaturation: 95 °C, 1 min<br>Annealing: 61 °C, 1 min<br>Extension: 72 °C, 1 min<br>Final extension: 72 °C, 5 min	30	1024
aflR	Initial denaturation: 95 °C, 1 min<br>Denaturation: 95 °C, 1 min<br>Annealing: 67 °C, 1 min<br>Extension: 72 °C, 1 min<br>Final extension: 72 °C, 5 min	30	1032
aflS	Initial denaturation: 95 °C, 10 min<br>Denaturation: 95 °C, 50 s<br>Annealing: 58 °C, 50 s<br>Extension: 72 °C, 2 min<br>Final extension: 72 °C, 5 min	30	684

Table S3. The content of IAA secreted by strain MR-86 and siderophores

Strain	IAA (μg/mL)	Synthesize siderophores (%)
MR-86	18.23	92.07

Table S4. Cohen’s d and 95% confidence interval (CI) for plant growth parameters

plant growth parameters	Cohen’s d	95% CI
Plant height	2.113	0.566 to 3.66
Root length	−0.078	−1.318 to 1.162
Root thickness	0.097	−1.143 to 1.337
Fresh weight per Plant	0.19	−1.052 to 1.432
Root freshness weight	0.277	−0.969 to 1.523
Dry weight	2.375	0.756 to 3.994

Table S5. Cohen's d and 95% confidence interval (CI) for soil chemical properties and enzyme activities

Chemical properties and enzyme activities	Cohen's d	95% CI
pH	3.425	0.912 to 5.938
organic matter	−2.135	−4.14 to 0.13
Alkaline-hydrolyzable nitrogen	4.238	1.355 to 7.121
Available p	12.867	5.413 to 20.321
Available K	1.909	−0.022 to3.84
Available Cu	0.626	−1.013 to 2.265
Available Zn	−0.911	−2.592 to 0.77
Available Fe	1.861	−0.055 to 3.777
Available Mn	−3.071	−5.433 to −0.709
Protease activity	8.311	3.344 to 13.278
Catalase activity	2.012	0.048 to 3.976
Phosphatase activity	0.453	−1.168 to 2.074
Urease activity	8.111	3.251 to 12.971
Cellulase activity	6.688	2.579 to 10.79
Sucrase activity	1.948	0.005 to 3.89

Table S6. Valid sequences and ASV numbers of bacteria and fungi were obtained from rhizosphere soil samples.

Group	bacteria		fungi	
	Raw_Tags	ASV	Raw_Tags	ASV
CK1	81623	4004	85300	622
CK2	85048	3576	84610	613
CK3	86780	3556	83817	691
CK4	87659	3905	81509	766
CK5	84542	3791	83509	769
MR-861	84546	3474	83640	708
MR-862	85439	3813	87864	784
MR-863	85587	3539	82167	731
MR-864	81838	3707	80212	665
MR-865	87961	3969	84284	699

Table S7. Probable and highly probable functional guilds

Functional guild names	Confidence level
Plant Pathogen	Probable
Animal Pathogen	Probable
Wood Saprotroph	Probable
Soil Saprotroph	Probable
Litter Saprotroph	Probable
Fungal Parasite	Probable
Arbuscular Mycorrhizal	Highly Probable
Dung Saprotroph	Probable
Ectomycorrhizal	Highly Probable
Endomycorrhizal	Highly Probable
